# *SCN5A* Variants: Association With Cardiac Disorders

**DOI:** 10.3389/fphys.2018.01372

**Published:** 2018-10-09

**Authors:** Wenjia Li, Lei Yin, Cheng Shen, Kai Hu, Junbo Ge, Aijun Sun

**Affiliations:** ^1^Department of Cardiology, Shanghai Institute of Cardiovascular Disease, Zhongshan Hospital, Fudan University, Shanghai, China; ^2^Department of Urology, Shanghai Tenth People's Hospital, Tongji University, Shanghai, China; ^3^Department of Cardiology, The Affiliated Hospital of Jining Medical University, Jining, China; ^4^Department of Cardiology, Institute of Biomedical Science, Fudan University, Shanghai, China

**Keywords:** *SCN5A*, Na_**v**_1.5, cardiac disorders, cardiac sodium channelopathy, therapeutic potential

## Abstract

The *SCN5A* gene encodes the alpha subunit of the main cardiac sodium channel Na_v_1.5. This channel predominates inward sodium current (INa) and plays a critical role in regulation of cardiac electrophysiological function. Since 1995, *SCN5A* variants have been found to be causatively associated with Brugada syndrome, long QT syndrome, cardiac conduction system dysfunction, dilated cardiomyopathy, etc. Previous genetic, electrophysiological, and molecular studies have identified the arrhythmic and cardiac structural characteristics induced by *SCN5A* variants. However, due to the variation of disease manifestations and genetic background, impact of environmental factors, as well as the presence of mixed phenotypes, the detailed and individualized physiological mechanisms in various *SCN5A*-related syndromes are not fully elucidated. This review summarizes the current knowledge of *SCN5A* genetic variations in different *SCN5A*-related cardiac disorders and the newly developed therapy strategies potentially useful to prevent and treat these disorders in clinical setting.

## Introduction

The *SCN5A* gene encodes the alpha subunit of the main cardiac sodium channel Na_v_1.5, which is known to be responsible for maintaining the normal function of inward sodium current (INa). INa current is the main component in fast depolarization phase after which the excitation–contraction coupling cascade and proper conduction of the electrical impulse is subsequently initiated within the heart (Aronsen et al., 2013).

Genetic variants of *SCN5A* are involved in a number of inherited cardiac channelopathies including Brugada syndrome (BrS), long QT syndrome (LQT3), and cardiac conduction system dysfunction. Meanwhile, *SCN5A* variants are also found to be correlated with myocardial contractile dysfunction, dilated cardiomyopathy, and heart failure. According to one of the most complete open database of on-line disease-related genetic variations “ClinVar” (ClinVar, 2018), more than 700 *SCN5A* variation locations are shown to be associated with cardiac disorders (Table 1) and about 90% variants account for non-synonymous variants, while the rest are due to deletion and duplication. With the help of patch clamp technique and transgenic animal technology, studies have clarified the major pathogenic mechanisms underlying sodium channelopathies due to *SCN5A* variants.

**Table 1:** Variants in *SCN5A* associated with cardiac disorders.

**Protein domain**	**Nucleotide change**	**Protein change**	**Biophysical properties of variant protein**	**Involvement in disease**	**References**
**LOCATED IN THE VSDs**					
DIII/S2	c.3745T>C	F1250L	Drug-induced LQT syndrome	LQT3	Yang et al., 2002
DIII/S3	c.3823G>A	D1275N	Associates with polymorphisms in the regulatory region of GJA5; decreases expression at the cell membrane; alters channel kinetics; shifts activation or inactivation state	DCM	Groenewegen et al., 2003; McNair et al., 2004; Meregalli et al., 2009; Kapplinger et al., 2010
DIII/S3	c.3890C>T	P1298L	No data	SSS	Benson et al., 2003
DIII/S4	c.3883G>A	E1295K	Causes significant positive shifts in the half-maximal voltage of steady-state inactivation and activation	LQT3	Abriel et al., 2001
DIV/S3	c.4783G>A	D1595N	Significant defects in the kinetics of fast-channel inactivation distinct from mutations reported in LQT3	PCCD;BrS	Wang et al., 2002
DIV/S4	c.4886G>A	R1629Q	Changes voltage-gated sodium channel activity; no difference in current density but changes inactivation kinetics and prolongs recovery from inactivation	BrS	Kapplinger et al., 2010; Zeng et al., 2013
DIV/S6	c.5302A>G	I1768V	Increases the rate of recovery from inactivation and the channel availability; as a positive shift of the steady-state inactivation curve	LQT3;BrS	Rivolta et al., 2002
**LOCATED IN THE LOOPS**					
D1 S3-S4 loop	c.1007C>T	P336L	Detected in a compound heterozygote also carrying V-1660; the presence of both mutations is necessary for the phenotypic expression of the disease; severe reduction of sodium currents	BrS	Cordeiro et al., 2006; Kapplinger et al., 2010
DI S5-S6 loop	c.892G>A	G298S	Reduces the whole cell current density and a delay in channel activation kinetics without a change in single-channel conductance	PCCD; Atrioventricular block; DCM	Wang et al., 2002; Saito et al., 2009
DIV S5-S6 loop	c.5111T>C	F1705S	Causes hyperpolarizing shift of steady-state inactivation and delays recovery from inactivation	SIDS	Otagiri et al., 2008
DIV S5-S6 loop	c.5126C>T	T1709M	No data	BrS;VF	Akai et al., 2000
DI-DII loop	c.1535C>T	T512I	Voltage-dependent activation and inactivation of the I-512 channel is shifted negatively by 8 to 9 mV and enhanced slower activation and slower recovery from inactivation compared to the wild-type channel; the double mutant R-558/I-512 channel shows that R-558 eliminates the negative shift induced by I-512 but only partially restores the kinetic abnormalities	PCCD;BrS	Viswanathan et al., 2003
DII-DIII loop	c.2893C>T	R965C	Steady state inactivation shifted to a more negative potential; slower recovery from inactivation	BrS	Priori et al., 2002; Hsueh et al., 2009; Kapplinger et al., 2010
DII-DIII loop	c.2989G>A	A997S	Also found in patients with atrial fibrillation; characterized by slower decay and a 2- to 3-fold increase in late sodium current	SIDS;BrS; LQT3	Ackerman et al., 2001; Darbar et al., 2008; Kapplinger et al., 2009
DII-DIII loop	c.3157G>A	E1053K	Abolishes binding to ANK3 and prevents accumulation of *SCN5A* at cell surface sites in ventricular cardiomyocytes	BrS;AF;LQT3	Priori et al., 2002; Mohler et al., 2004; Darbar et al., 2008; Kapplinger et al., 2009, 2010
DII-DIII loop	c.3250G>C	G1084S	Rare polymorphism	SIDS	Otagiri et al., 2008
DIII-DIV loop	c.4531C>T	R1512W	Significantly affects cardiac sodium channel characteristics; associated with an increase in inward sodium current during the action potential upstroke	Primary familial hypertrophic cardiomyopathy;BrS	Rook et al., 1999; Smits et al., 2002; Meregalli et al., 2009
C-terminus	c.5381A>G	Y1795C	Slows the onset of activation, but does not cause a marked negative shift in the voltage dependence of inactivation or affect the kinetics of the recovery from inactivation; increases the expression of sustained Na^+^ channel activity and promotes entrance into an intermediate or slowly developing inactivated state	LQT3	Rivolta et al., 2001; Tester et al., 2005; Benito et al., 2008; Kapplinger et al., 2009
C-terminus	c.5474G>A	R1826H	Characterized by slower decay and a 2- to 3-fold increase in late sodium current	LQT3;SIDS; BrS	Ackerman et al., 2001; Kapplinger et al., 2009
C-terminus	c.5546A>G	H1849R	Decreases interaction with FGF12, FGF13 and FGF14; increases voltage-gated sodium channel activity; alters inactivation	LQT3;BrS	Musa et al., 2015
C-terminus	c.5708C>T	S1904L	Promotes late sodium currents by increasing the propensity of the channel to reopen during prolonged depolarization	LQT3;BrS	Bankston et al., 2007
N-terminus	c.128G>A	R43Q	Does not affect baseline kinetics of sodium currents; causes an unusual hyperpolarizing shift of the activation kinetics after lidocaine treatment	LQT3; BrS	Lilet et al., 1991; Kapplinger et al., 2009

*Thousands of SCN5A variants have been detected and most of them are considered to be closely related to human diseases. More than one hundred variants in the SCN5A gene have been described leading to different types of cardiomyopathies according to the existing resources on ClinVar, OMIM and UniProt which provide the comprehensive, authoritative compendium of human genes and genetic phenotypes. Here, 22 variants are listed as they were observed in at least one specific cardiac disorders. PCCD, Progressive Cardiac Conduction Defect; DCM, Dilated Cardiomyopathy; BrS, Brugada Syndrome; LQT3, Long-QT Syndrome; SIDS, Sudden Infant Death Syndrome; AF, Atrial Fibrillation; SSS, Sick Sinus Syndrome; VF, Ventricular Fibrillation*.

Recent research results indicated that the function and regulation of Na_v_1.5 was more complicated than traditionally assumed. For example, Na_v_1.5 has been found to be regulated by more than 20 interacting proteins in distinct membrane compartments (Shy et al., 2013). The dysfunction of Na_v_1.5 may not only be a cause but also a consequence in different pathophysiological procedures and various cardiac disorders. Undoubtedly the dysfunction of Na_v_1.5 contributes to arrhythmogenesis during pathophysiological conditions. However, sodium channel remodeling manifested with alterations in Na_v_1.5 clustering was also discovered within distinct cardiomyocyte microdomains in some pathophysiological procedures such as heart failure (Rivaud et al., 2017). We previously found that the *SCN5A* variant A1180V could directly lead to cardiac structural impairment without involvement of long-time arrhythmias (Shen et al., 2013). Similarly, the environmental and genetic factors may also play additional roles in pathogenesis of disorders linked to *SCN5A* variants.

In this review, we will summarize *SCN5A* variants-related arrhythmia syndromes, structural cardiac disorders and the underlying potential mechanisms. Then a brief description will also be given to introduce the current knowledge regarding the therapy strategies.

## Structure and function of *SCN5A*

**Figure 1:**
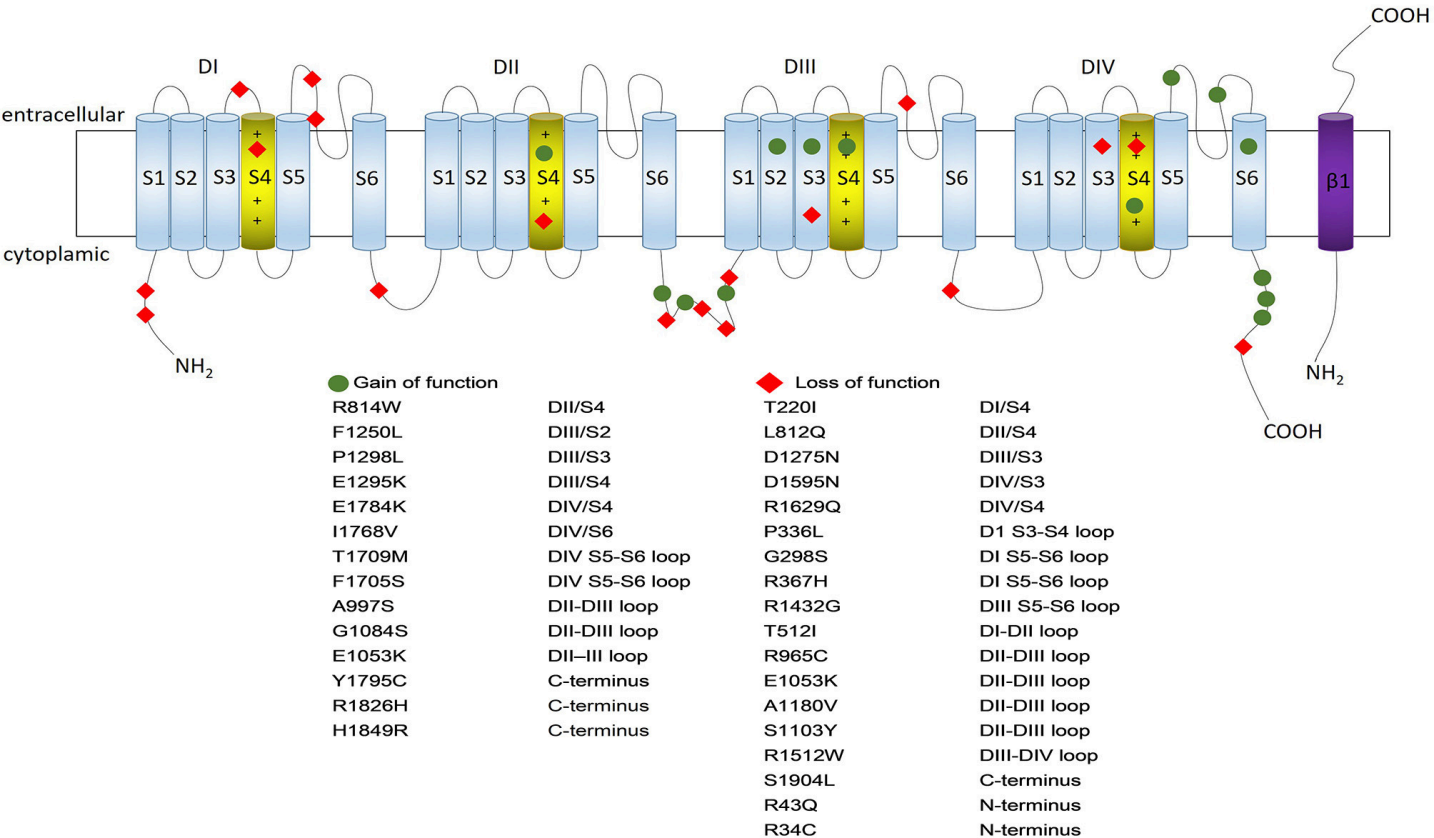
Na_v_1.5 protein structure and some typical variants associated with gain- or loss- of the sodium channel function. Na_v_1.5 is a large transmembrane protein with four internally homologous domains (DI-DIV), each containing six spanning segments (S1–S6) which are indicated by numbered cylinders. The four domains are interconnected by intracellular peptide chains, with N-terminal and C-end both located in the intracellular side. S4 segment works as a critical voltage-sensor because of its ample positive charge residues; S5 and S6 segments are lined by extracellular loops (P-loops) which are considered to determine the ion selectivity of the channel (S4 segments are depicted in yellow while the rest segments being blue). The transmembrane segment resembles one of the beta-subunits, which is depicted in purple. The location of the gain-of-function *SCN5A* variants are shown in blue circle while location of the loss-of-function *SCN5A* variants are shown in red rhombus.

The *SCN5A* gene, located in chromosome 3p21 with 28 exons, is a member of the human voltage-gated sodium channel gene family and encodes alpha subunit of the main cardiac sodium channel Na_v_1.5. It was firstly identified by Georgè in 1995 with the help of fluorescence *in situ* hybridization (George et al., 1995). Na_v_1.5 is a large transmembrane protein (227 KDa) with four internally homologous domains (DI-DIV), each containing six transmembrane spanning segments (S1-S6) (Figure 1). S4 segment works as a critical voltage-sensor because of its ample positive charge residues. It is triggered to undergo transmembrane movement when the cell membrane depolarizes, allowing for sodium ion influx to generate sodium current. The P-loops between S5 and S6 segments constitute the central pore forming region, which determine the ion selectivity of the channel. The four domains are interconnected by intracellular peptide chains, with N-terminal and C-end both located in the intracellular side (Catterall, 2014; Chen-Izu et al., 2015). Previous studies suggested the *SCN5A* gene was mainly expressed in cardiomyocytes, however, recent researches found this gene also expressed and played fundamental roles in other tissues such as brain, gastrointestinal (GI) tract, and cancer tissues (Black and Waxman, 2013; Verstraelen et al., 2015).

There are two major *SCN5A* isoforms: “adult” isoform (*SCN5A*-003, NM_000335) and “neonatal” (or fetal) isoform (*SCN5A*-001, NM_001099404). The “adult” *SCN5A* isoform is abundantly expressed in adult human heart while the “neonatal” isoform is abundantly expressed in neonatal heart. The “adult” isoform differs from the “neonatal” isoform in exon 6 within a voltage-sensor domain (D1/S3-S4) (Murphy et al., 2012). In a mutually exclusive splicing manner, the “neonatal” isoform exhibited slower kinetic of activation and inactivation and a lower depolarized threshold of activation compared to the “adult” isoform (Onkal et al., 2008). The latest study found that both fetal (exon 6a) and adult (exon 6) isoforms of *SCN5A* were expressed after short-term culture of human induced pluripotent stem cell-derived cardiomyocytes (hiPSC-CMs) with one of *SCN5A* variants I230T. Interestingly, prolonged culture time increased the expression of adult sodium channel isoform, which was paralleled by a decrease in INa, representing a severe clinical phenotype in patients with recessive cardiac conduction disease (Veerman et al., 2017). This result partly explained the electrophysiological immaturity phenomenon of hiPSC-CMs and refined our understanding on pathophysiological mechanism of a recessive form of *SCN5A*-related conduction disorder. Apart from these two isoforms, *SCN5A*-014 (NM_198056) is also expressed in human heart and should be mentioned in some detail. *SCN5A*-014 includes an additional glutamine at position 1077 (1077Q) at the exon 17–18 splice boundary, resulting in smaller INa current and exhibiting functional differences with the previous standard isoforms (Makielski et al., 2003).

Sodium current participates in phase 0 and 2 of action potential (AP), which is an important determinant on the production of heart rhythms, normal conduction, and maintenance of excitement. Aberrant sodium channels resulting from *SCN5A* variants would potentially cause disorganization of the cardiac electrophysiological system, produce various arrhythmias and result in structural heart diseases. Inward and outward transmembrane currents are in dynamic balance physiologically, while variants in *SCN5A* could induce two states in the sodium channel: “gain-of-function state” or “loss-of-function state.” The former state appears as an increase in sodium influx (sodium current) and a delay in inactivation, while the latter one is manifested by a decrease in sodium influx and an acceleration of inactivation. Different variants-related phenotypes correspond to different state.

## Cardiac arrhythmia

### Sick sinus syndrome (SSS)

Sick sinus syndrome (SSS) is characterized by dysfunction of the sinus node. SSS patients exhibit inappropriate sinus bradycardia, sinus arrest, and reduced chronotropic response (Abe et al., 2014). Although the disease usually affects elderly patients with underlying structural cardiac abnormalities, such as fibrosis or ischemia, young hereditary SSS patients are also not rare (John and Kumar, 2016). One of the genes linked to hereditary SSS is *SCN5A* and 128 *SCN5A* variants were found to be causative of hereditary SSS by the complete sequencing of the human genome (ClinVar, 2018). Benson's study firstly discovered six compound heterozygous variants of *SCN5A* and defined *SCN5A*-related SSS was a “recessive” disorder often exhibiting compound heterozygosity for distinct *SCN5A* alleles which associated with dominant disorders of cardiac excitability (Benson et al., 2003). Current evidences indicated that cardiac excitability would decrease and sinus node became dysfunctional in case of both lost or enhanced sodium channel function induced by *SCN5A* variants. The most typical state of *SCN5A* variants is the loss-of-function property with reduced electric coupling between sinus node and surrounding atrial cells, resulting in the exit block, a common feature in SSS (Abe et al., 2014). *SCN5A* variants with gain-of-function feature could induce an increase in AP duration as a result of persistent current during the systolic phase. The prolongation of the sinus node AP would prolong the cycle length, thereby reduced the sinus rate (Wilders, 2018). The phenotypic and functional changes in sinus node caused by *SCN5A* variants have been carefully observed, however, the electrical activity and the underlying regulation mechanisms have not been extensively studied. Previous studies found that heterozygous *SCN5A* variant genotype was associated with inappropriate sinus bradycardia, impaired atrioventricular conduction, or increased ventricular refractoriness, while homozygous genotype resulted in intrauterine lethality (Papadatos et al., 2002). However, a recent case report challenged this viewpoint. Jenewein et al. (2017) studied a family with E1053K variant and found that the phenotypical expression in carriers of the same E1053K variant ranged from asymptomatic phenotype to sudden cardiac death (SCD), some asymptomatic individuals were even homozygous variant carriers. Moreover, there are some signaling pathways that regulate the expression and function of *SCN5A*. Notch signaling is a signaling pathway that can be transiently activated in response to various cardiomyocytes injuries. It was discovered that a transient Notch pulse could down-regulate *SCN5A* expression, which might result in a slowed atrial conduction velocity (Qiao et al., 2017). As *SCN5A* variants are associated with large phenotypic variability, future studies are warranted to understand the alternations of electrical activity in mutant pacemaker cells.

### Atrial arrhythmias

Atrial arrhythmias associated with *SCN5A* variants mostly include familial atrial standstill and atrial fibrillation. Atrial standstill (AS) is caused by atrial electrical and mechanical impairment, manifested by disappeared P wave, bradycardia, or borderline escape rhythm (Baskar et al., 2014). Missense variants such as R367H caused electrophysiological disorders with a non-functional Na^+^ channel, which could finally lead to AS and BrS (Takehara et al., 2004). Other variant types such as frameshift variants or compound heterozygous variants were also predicted to result in loss-of-function sodium channel and induce supraventricular arrhythmia in patients with AS (Baskar et al., 2014). Groenewegen et al. (2003) performed genetic screening of *SCN5A* and other atrial-specific genes in an apparently sporadic case of AS and found *SCN5A* D1275N variant in a familial AS pedigree. This variant resulted in a depolarizing shift in activation. Interestingly, D1275N was found to be closely linked to the atrial-specific gap junction protein connexin40 (Cx40). The decreased expression of Cx40 jointly impaired the electrical coupling between atrial cells. It was thus speculated that genetic variants could synergistically enhance the functional effect of each individual genetic change, consequently leading to a complete loss of electrical and mechanical activity.

Atrial fibrillation (AF) is the most common sustained arrhythmia characterized by rapid and irregular beating of the atria (Hucker et al., 2016). A Framingham study showed that the incidence of new-onset AF was 26.8% after a 9-year follow-up period. Besides, new-onset AF occurred more frequently among individuals with familial history than without familial AF history, indicating that the hereditary factors might play an important role in AF pathogenesis (Lubitz et al., 2010). To date, 32 genes including *SCN5A* have been shown to be associated with AF (Hayashi et al., 2017). A clinical study scanned *SCN5A* variants in 156 unrelated probands and AF was evidenced in 43% individuals with *SCN5A* variants (Olson et al., 2005). Another study also reported a high prevalence of *SCN5A* variants in familial AF patients (Darbar et al., 2008). Moreover, there was a high overlap between AF and other *SCN5A* variants related diseases i.e., LQT3, BrS, SSS, and conduction disease (Savio-Galimberti and Darbar, 2014; Ishikawa et al., 2017; Boddum et al., 2018). These emerging evidences suggested a possible link between variants in *SCN5A* and familial AF. Both gain-of-function and loss-of-function variants in *SCN5A* have been described to induce atrial arrhythmia. Gain-of-function variants such as D1275N promoted ectopic activity and increased atrial AP duration and excitability, while loss-of-function variants such as H558R brought a reduction in sodium current density and a shortening refractory period, then decreased conduction velocity in the atria and finally increased vulnerability to atrial arrhythmia.

### Ventricular arrhythmias

Variants of *SCN5A* have been demonstrated to contribute to various kinds of ventricular arrhythmias including long QT syndrome (LQTS) type 3; Brugada syndrome (BrS) and idiopathic ventricular fibrillation (IVF), which greatly increase the risk of sudden death in young individuals with a structurally normal heart. Decades of researches have raised enormous insights into the role of *SCN5A* variants in ventricular arrhythmogenesis.

#### Long-QT syndrome type 3(LQT3)

Long-QT syndrome (LQTS) is an inherited arrhythmogenic disease characterized by prolonged QT intervals. Among the 15 current forms of LQTS, long QT syndrome type 3 (LQT3) is caused by inherited variants of *SCN5A* accompanied with an increasing risk to sudden death during rest or sleep and electrocardiographically characterized by prolonged QT/QTc interval, accentuated QT dispersion, late onset of T wave and frequent prominent U wave. The first description of LQT3 variant was presented in 1995 with a deletion of amino acids 1,505–1,507 (ΔKPQ) in a patient with prolonged QT interval (Wang et al., 1995a). Up to now, over 300 *SCN5A* variants are known to be related to LQT3. Five to ten percentage of LQT3 patients possess gain-of-function variants in *SCN5A* such as E1784K, E1053K, I1768V etc. (Flaim et al., 2007; Remme, 2013; Veerman et al., 2015; Jenewein et al., 2017). These variants are mostly located in the intracellular junctions between DIII and DIV region, S4 segment and the C-terminal region, which could cause the channel inactivation disorders, persistent sodium channel opening state, inactivation of inward sodium current, and prolonged AP (Wang et al., 1995b). The current alternation disturbed the balance between ion influx and outflux in the plateau phase of the AP, which could result in a fast AP initiation and late inward Na^+^ current (INaL), thereby easily induced Torsades de pointes (TdP) and ventricular fibrillation (VF) (Fredj et al., 2006). In addition, intracellular calcium homeostasis and Ca^2+^ transient amplitude would influence window current or INaL, as a result, the mixed syndrome phenotype in LQT3 patients would ameliorated or exacerbated in the setting of a *SCN5A* variants (Rivaud et al., 2018). The predisposition to VF is also influenced by genetic modifiers. Ter Bekke and his colleagues phenotyped a 16-generation pedigree and found *SCN5A*-p.Phe1617del carriers were predominantly female family members, characterized with after arousal-evoked heart-rate acceleration and repolarization prolongation (Ter Bekke et al., 2017). Besides, many variants were associated with more than one arrhythmia phenotypes at the same time (Kimura et al., 2017). The most common *SCN5A* variant E1784K was not only underpinning both Brugada syndrome type 1 (BrS1) and LQT3, but being influenced by both elevated temperature and cytosolic calcium (Abdelsayed et al., 2018). The interrelationships and regulatory mechanisms of *SCN5A* variants are so complicated that there is still a long way to go to pinpoint various pathogenic mechanisms underlying the various arrhythmia syndrome.

#### Brugada syndrome

Brugada syndrome (BrS) is a familial arrhythmia syndrome characterized by ventricular arrhythmias and SCD, in which the signature feature of type-I BrS ECG pattern is ST-elevations in the right precordial leads (Sieira and Brugada, 2017). It often occurs in healthy individuals at a relatively young age (< 40 years). In BrS, the first variant was identified in an α-subunit of the sodium channel gene, *SCN5A* in 1998 (Chen et al., 1998). A study examined the genotype-phenotype correlation of *SCN5A* variants in BrS with a 72 months followup period and found that BrS patients with *SCN5A* variants exhibited more conduction abnormalities and had higher risk for cardiac events (Yamagata et al., 2017). To date, nearly a quarter of BrS patients were found to be *SCN5A* variants carriers and over 300 *SCN5A* variants were found to be associated with BrS, which include missense variants, non-sense variants, nucleotide insertion/deletions, and splice site variants (Kapplinger et al., 2010). BrS-related *SCN5A* variants are usually loss-of-function variants and mainly located in the region between DI and DII, intracellular connection between DIII and DIV region, P ring and D-terminal of DIII region including *SCN5A* polymorphism H558R, R34C, S1103Y, L812Q, K817E, etc. (Kapplinger et al., 2010; Wang et al., 2015; Kinoshita et al., 2016; Matsumura et al., 2017). These variants disturbed the transmembrane ion flux balance at the end of the first phase of AP. As a result, there appeared notch on the epicardial AP, which presented ST segment elevation on ECG. When the current imbalance increased, the epicardial AP would become significantly shorter and the phase 2 bipolar state would become unbalanced with reentry, leading to the occurrence of ventricular tachycardia and ventricular fibrillation (Grant et al., 2002). In addition to the abnormal AP duration restitution properties, conduction delay in the right ventricle outflow tract were also observed in BrS patients. The depolarization disorder hypothesis mostly derived from clinical observations (Takami et al., 2003; Tukkie et al., 2004). The delayed and fragmented ventricular conduction was thought to be one of the potent arrhythmogenic substrates as strong predictors of ventricular arrhythmias in patients with BrS (Kanda et al., 2002; Nishii et al., 2010).

#### Idiopathic ventricular fibrillation

Idiopathic ventricular fibrillation (IVF) is characterized by spontaneous ventricular fibrillation and exclusion of any specific structural or functional cardiac diseases including structural cardiac diseases (i.e., coronary artery disease, valvular heart disease, myocarditis, hypertrophic, and dilated cardiomyopathy) and primary arrhythmia syndromes [i.e., BrS, long- and short-QT syndromes, catecholaminergic polymorphic ventricular tachycardia (CPVT), and early repolarization syndrome (ERS)] (Priori et al., 2013). Truncated, non-functional Na^+^ channel α-subunit resulting from *SCN5A* variants was found to be one of the underlying causes for IVF including missense variants such as R1432G, R1512W, A1924T, and insertion variants such as 1795insD (Chen et al., 1998). Some studies indicated that IVF variants of *SCN5A* reduced Na^+^ channel function and decreased the availability of Na^+^ conductance, however, the mutant phenotype became different with experimental conditions changes (e.g., presence of β1-subunits; temperature) as well as variable region location (Wan et al., 2001; Maltsev et al., 2009). The exact pathogenesis and pathophysiological mechanism of IVF are unknown. The origin might be mono- or polygenic variants and may be multifactorially influenced by particular environmental or discrete structural abnormalities.

### Cardiac conduction defect

Progressive cardiac conduction defect (PCCD), also called Lenegre syndrome or Lev disease, is one of the most common conduction disorders. It is characterized by progressive changes in the His-Purkinje system with either right or left bundle branch block and QRS complex broadening, eventually leading to complete atrioventricular block, syncope or sudden death. Variants in *SCN5A* as a cause of inherited PCCD were firstly described in 1999 (Schott et al., 1999). *SCN5A* variants led to a decrease in sodium channel density on cell membrane, as a result, the decreased current in phase 0 slowed down the myocardial conduction speed and eventually resulted in various conduction blocks. Considerable overlaps existed between PCCD and other clinical entities such as BrS and LQT3 (Glaaser et al., 2012; Park et al., 2015; Veltmann et al., 2016). Similar to BrS, loss-of-function *SCN5A* variants could decrease sodium channel availability and led to PCCD, while gain-of-function *SCN5A* variants underlying LQT3 could prolong AP and increased persistent inward sodium, which might also occur in PCCD. Some pathogenic variants of *SCN5A* associated with cardiac conduction disturbances might be a genetic marker and a non-negligible alarm signal associated with ventricular arrhythmia and SCD (Makarawate et al., 2017). Therefore, proper follow-up of *SCN5A* variants carriers with PCCD phenotype is warranted.

## Dilated cardiomyopathy and heart failure

Dilated cardiomyopathy (DCM) is a myocardial disease characterized by left ventricular systolic dysfunction with dilatation of left ventricle or double ventricles. It is the most common type of primary cardiomyopathy and the most common reason for cardiac transplantation in adults and children (Sanbe, 2013). It was reported that 20–50% DCM patients had a family history, suggesting that genetic factors played an important role in their pathogenesis. Currently, more than 40 causative genes have been found in DCM, including *SCN5A* (Hershberger et al., 2013). In 2004, Mc Nair firstly identified a heterozygous G-to-A variant of *SCN5A*, which was co-segregated with DCM phenotype (McNair et al., 2004). Subsequently, *SCN5A* missense variants (T220I, R814W, D1595H) and truncation variants (2550-2551insTG) were successively discovered in DCM patients (Olson et al., 2005). Most variants are located in the voltage sensor domain (VSD) especially in S3 and S4 segment. As the electrophysiological properties of these variants are not fully clarified, the pathogenesis of DCM due to *SCN5A* variants remains inconclusive.

Systolic function of the heart was previously thought to be mainly associated with calcium channels. *SCN5A* variants were traditionally recognized as the cause of arrythmias in DCM patients, but did not directly affect the cardiac systolic function. A primary disruption of an ion channel through gene variants leading to DCM was explained that in one way, the ion channel disturbance caused dysfunction of cytoskeletal protein binding partners and resulted in a DCM phenotype. In another way, the electrical dysfunction caused by variants defects led to mechanical instability, and ultimately led to myocardial dilation (Towbin and Lorts, 2011). Furthermore, gain-of-function *SCN5A* variants were partly responsible for hyperexcitability of the fascicular-Purkinje system. The incomplete repolarization in Purkinje cells brought premature ventricular action potentials and developed DCM finally (Laurent et al., 2012).

Through the electrophysiological analysis of A1180V, a novel *SCN5A* variant that was discovered by our team, we indicated that variant channel expressed a rate-dependent Na^+^ current reduction and a moderate increase in INaL, implying that A1180V caused DCM by disturbing cellular Na^+^ homeostasis. During the follow-up period of the affected pedigree, we found A1180V carriers exhibited a deterioration of cardiac function and progressed to DCM or atrioventricular block (AVB). These results suggested that A1180V was a risk factor for familiar DCM with preceding atrioventricular block (Ge et al., 2008; Shen et al., 2013). The variant might directly damage myocardium by affecting the intracellular sodium homeostasis, which in turn lead to cell membrane Na^+^/Ca^2+^- and Na^+^/H^+^-exchanger disorders and imbalance of intracellular calcium homeostasis. Apart from A1180V, we further explored three novel non-synonymous *SCN5A* variants in idiopathic DCM patients, including c.674G>A, c.677C>T, and c.4340T>A (Shen et al., 2017). Consistent with previous results, these newly defined idiopathic DCM related variants were mainly located in the S4 segment of domain I (DI-S4). By applying patch clamp technology, we found that R225Q (c.674G>A) predisposed electrical disorders by reducing peak sodium current density. Some Na_v_1.5 variants such as R222Q; R225W; R225P; R814W; and R219H were associated with an atypical phenotype combining several cardiac arrhythmias and DCM (McNair et al., 2011; Mann et al., 2012). Chahine's group investigated mutant channels and found the gating pore current generated by these variants with a cation leak through the typically non-conductive VSD. The presence of a proton-based leak current linked Na_v_1.5 VSD variants with human cardiac arrhythmias and dilatation of cardiac chambers (Gosselin-Badaroudine et al., 2012; Moreau et al., 2015a,b). As many of the molecular mechanisms between the dilated phenotype and DCM-related *SCN5A* variants have yet to be elucidated, future novel insights will be available depending on transgenic mice studies and large-scale clinical long-term follow-up studies.

## Sudden infant death syndrome

Sudden infant death syndrome (SIDS) denominates cases of infant death, which mostly occurs during sleep without any preceding symptoms. It is the leading cause of post neonatal mortality (Matthews and Macdorman, 2013). Although many pathophysiological studies have been proposed, the specific pathogenic mechanisms remain unclear. SIDS is presumed to be co-regulated by multiple factors including neurotransmission, energy metabolism and genetic disorders. Ion channel genes such as *KCNQ1, KCNH2, SCN5A, RYR2*, and their molecular modifiers such as GPD1L and β-subunits account for ~10–15% of all SIDS cases, in which *SCN5A* variants account for approximately half of the channelopathic SIDS cases (Tan et al., 2010; Tester et al., 2018). Variants in *SCN5A* led to severe trafficking defects and a severely dysfunctional Na_v_1.5 channel in a structurally normal heart. The decreased current density was likely to be a major contributing factor to the lethal arrhythmia in SIDS victim (Gando et al., 2017). Given the potential risk of inherited cardiac conditions caused by ultra-rare gene variants, current experts has made consensus statement on post-mortem genetic testing (molecular autopsy) and clinical investigation of surviving blood relatives. However, under pressure from public moral principle and different physical environments, roll-out of this approach seems a long way away.

## Arrhythmogenic right ventricular cardiomyopathy

Arrhythmogenic right ventricular cardiomyopathy (ARVC) is an inherited cardiomyopathy characterized by myocardial atrophy with fibro-fatty replacement, eventually resulting in wall thinning and cardiac dilatation. The inflow tract, outflow tract and apex of the right ventricle constitute the so-called “triangle of dysplasia” (Oomen et al., 2018). ARVC manifests with myocardial inflammation, right ventricular aneurysms, and ventricular tachyarrhythmias, which represents one of the leading causes of sudden death in the young and in athletes (Basso et al., 2018). The estimated prevalence of ARVC is 1 per 5000 in the general population. Approximately >60% of patients have a pathogenic variant (Bhonsale et al., 2015; Calkins et al., 2017). Desmosomes variants was traditionally considered as the genetic basis of ARVC (Gandjbakhch et al., 2013). It was known that desmosomal protein desmoglein-2 and Na_v_1.5 interacted with each other (Rizzo et al., 2012). Decreased Na^+^ current density could slow down conduction and increase necrosis and fibrosis. An artificial loss of desmoplakin (DSP) expression induced an abnormal distribution of connexin43 (Cx43) and Na_v_1.5. As a result, the sodium current decreased and conduction velocity slowed down, which impaired the mechanical and electrical coupling, these factors also contributed to the pathogenesis of ARVC (Zhang et al., 2013). Beside these, Te Riele performed whole-exome sequencing in six ARVD/C patients without desmosomal variants and found a missense variant (R1898H) in *SCN5A*. Later, they expanded the study population and found almost 2% of ARVD/C patients had rare *SCN5A* variants (Te et al., 2017). These findings demonstrated that changes in sodium channel might interact with other molecules or form a tighter network of interactions and served as potential causal gene variants of ARVC.

## Overlap syndrome

Multiple *SCN5A* variants caused complex clinical phenotypes, which suggested that different arrhythmias might have the same genetic origin. The clinical and genetic overlap between these arrhythmias are considered overlap syndrome. 1795insD was the first *SCN5A* variant related to overlap syndrome, carriers not only presented with LQT3, but also with sinus bradycardia, PCCD, and BrS (Postema et al., 2009). Subsequently, similar findings were reported for other *SCN5A* variants such as DelK1500, E1784K, Q779X (Grant et al., 2002; Sumitomo, 2014; Aoki et al., 2017). Various *SCN5A*-related arrhythmia syndromes were not separated but closely linked clinical entities with numerous biophysical overlaps. Moreover, there was a strong genotype-phenotype correlation between arrhythmia syndromes and *SCN5A* variants, although some phenotypes presented during all decades of life, while others developed with increasing age. By observation in various heterologous expression systems, the biophysical characteristics underlying multiple phenotypes were found to delay fast inactivation, reduce peak sodium current density and increase late component of Ina (Remme and Wilde, 2008; Veltmann et al., 2016). The complex phenotype could be explained by the changes in membrane potential and current. Reduced penetrance and variable disease expression might limit the clinical and genetic diagnosis in *SCN5A*-related overlap syndromes. Even owning the same variant in *SCN5A*, reasons of the presence of different or even opposite phenotype are still unclear. The fact that the same *SCN5A* variant might have different phenotypes suggests the phenotype may have individual or family differences, or that certain regulatory genes or environmental factors may also affect the presence of phenotypes. In addition, clinical elements including age, gender, or medications may also modify disease expressivity. Large cohorts studies are thus needed to gain more insight into exact causal relationship between sodium channel function and various phenotypes.

## Potential therapeutic strategies

For many years, researchers have been working on reversing of the pathogenic phenotypes caused by *SCN5A* variants. Although implantation of ICDs can prevent potentially fatal arrhythmias in patients, the main stream of therapy is the use of medications to prevent arrhythmias. Classical sodium channel blockers are classified as Class I anti-arrhythmic agents and furtherly subdivide into classes IA, IB and IC according to their effects on changes of cardiac AP length (Milne et al., 1984). The blockade of sodium channels can reduce the excitability of cardiomyocytes and stop reentrant wavefronts. However, the same mechanism may exert opposing effect at the same time (van Hoeijen et al., 2014). Toxicities include proarrhythmia are common. Whether the sodium channel blocker mexiletine can be used in clinic to shorten the QT interval without side effects among *SCN5A* variants carriers has long been controversial. As early as 2004, it was found to rescue the membrane expression of the voltage-gated sodium channel and assist in transporting proteins from the sarcoplasmic reticulum to plasma membrane (Valdivia et al., 2004). Later research queried mexiletine might facilitate trafficking of mutant proteins and exacerbating QT prolongation (Ruan et al., 2010). However, recent study demonstrated mexiletine could bring a major reduction of life-threatening arrhythmic events in LQT3 patients in a retrospective cohort study (Mazzanti et al., 2016). Even with so many conflicting studies, selective inhibition of INaL constitutes a promising pharmacological treatment for cardiac channelopathies. Many pharmacists are dedicated to finding a potent, selective inhibitor of INaL. For example, the INaL inhibitor GS967 was discovered to decrease repolarization abnormalities and had anti-arrhythmic effect without damnification on cardiac conduction (Portero et al., 2017). Other traditional drugs such as flecainide, lidocaine, or β-blockers have also been reported to have some clinical benefits in certain population or specific variants (Wilde et al., 2016; Anderson et al., 2017; Chorin et al., 2018). In general, the especially important issue in pharmacological treatment lays in rebranding of old medications and development of novel agents on the basis of ascertainment in the molecular derangements resulting from genetic variants (Varian and Tang, 2017).

In addition to pharmacological treatments and ICD implantation, some other new approaches are gradually emerging. Non-sense variant is one type of *SCN5A* pathogenic variants. Non-sense mutation readthrough is a new kind of gene-specific treatment to reduce or avoid deleterious consequences of non-sense variants which could make sure the ribosomes ignore a premature stop codon and produce a full-length protein (Lee and Dougherty, 2012). Some researchers applied readthrough-enhancing methods such as aminoglycosides, suppressor tRNAs or small interfering RNAs (siRNAs) to suppress variants (Roy et al., 2016; Baradaran-Heravi et al., 2017). The sodium currents were restored, however, the restored channels increased the risk of arrhythmia (Teng et al., 2017). Pre-implantation genetic diagnosis (PGD) is a diagnostic procedure by analyzing genotype of the preimplantation embryo, enabling selective transfer of unaffected embryos to the uterus. This procedure improves embryo selection and avoids pathogenesis-related genetic variants. A meta-analysis including four randomized controlled trials (RCTs) and seven cohort studies showed that comprehensive chromosome screening (CCS)-based PGD has better outcomes for women compared to classical morphological criteria (Chen et al., 2015). Although the prospect is raising difficult ethical considerations, PGD is one of the strategies for prevention of gene variants-related diseases somehow (Resnik, 2014; Vermeesch et al., 2016). It is known that gene works through mRNA level. Some studies attempted to regulate the expression of *SCN5A* mRNA through exogenous ingestion of the messenger RNA (mRNA) stabilizing protein ELAVL1/HuR and to verify whether it was beneficial to control arrhythmia. Researchers found injection of viral particles carrying HuR increased *SCN5A* expression and improved AP upstroke and conduction velocity, which reduced reentrant arrhythmia. Increasing mRNA stability to rescue decreased *SCN5A* expression may represent another new paradigm in antiarrhythmic therapy (Zhou et al., 2018). Abnormal bioelectric phenotype was discovered to be corrected in cystic fibrosis caused by variants of the cystic fibrosis transmembrane conductance regulator gene (CFTR) by administering replication-deficient, recombinant adenovirus vector containing a normal copy of the CFTR cDNA (AdCFTR) in a clinical trial (Hay et al., 1995). But arrhythmia genetic or epigenetic therapy investigations have been limited to short-term demonstrations of efficacy in pre-clinical pilot studies. For many arrhythmia applications, adequate delivery to the target tissue and proarrhythmic toxicity as well as safety risks still remain. With long-term efficacy and safety studies, integrated approaches of normalizing cardiac structure and function may represent the next generation of potential therapeutic strategies for cardiac disorders with gene variants.

## Conclusions and perspectives

Cardiac sodium channel plays a central role in cardiomyocyte excitability and proper conduction of cardiac electrical impulses. The function and mechanisms of sodium channel are complicate and remain a lot of controversies. Even within families, the observed phenotypes carrying the same *SCN5A* variant are highly diverse. Besides, environmental and epigenetic alterations also determine variable disease severity. Continuous exploration and novel insight of this issue will contribute to clarify detailed mechanisms and ultimately enable improved diagnosis, risk stratification, and development of more effective treatment strategies.

## Author contributions

All authors listed have made a substantial, direct and intellectual contribution to the work, and approved it for publication. In particular, WL, LY, and CS: drafting of the manuscript; KH, JG, and AS: revising the manuscript for important intellectual content critically and for final approval of the manuscript.

### Conflict of interest statement

The authors declare that the research was conducted in the absence of any commercial or financial relationships that could be construed as a potential conflict of interest.
